# Age-Related Differences in the Cognitive, Visual, and Temporal Demands of In-Vehicle Information Systems

**DOI:** 10.3389/fpsyg.2020.01154

**Published:** 2020-06-03

**Authors:** Joel M. Cooper, Camille L. Wheatley, Madeleine M. McCarty, Conner J. Motzkus, Clara L. Lopes, Gus G. Erickson, Brian R. W. Baucom, William J. Horrey, David L. Strayer

**Affiliations:** Applied Cognition Lab, Department of Psychology, The University of Utah, Salt Lake City, UT, United States

**Keywords:** driving, reaction time, aging, technology, attention, workload

## Abstract

In-vehicle information systems (IVIS) refer to a collection of features in vehicles that allow motorists to complete tasks (often unrelated to driving) while operating the vehicle. These systems may interfere, to a greater extent, with older drivers’ ability to attend to the visual and cognitive demands of the driving environment. The current study sought to examine age-related differences in the visual, cognitive and temporal demands associated with IVIS interactions. Older and younger drivers completed a set of common tasks using the IVIS of a representative sample of six different vehicles while they drove along a low-density residential street. Evaluation measures included a Detection Response Task (DRT), to assess both cognitive and visual attention, and subjective measures following each condition using the NASA Task Load Index (TLX). Two age cohorts were evaluated: younger drivers between 21 and 36 years of age, and older drivers between 55 and 75 years of age. Participants completed experimental tasks involving interactions with the IVIS to achieve a specific goal (i.e., using the touch screen to tune the radio to a station; using voice commands to find a specified navigation destination, etc.). Performance of tasks varied according to different modes of interaction available in the vehicles. Older drivers took longer to complete tasks, were slower to react to stimuli, and reported higher task demand when interacting with IVIS. Older drivers stand to benefit the most from advancements in-vehicle technology, but ironically may struggle the most to use them. The results document significant age-related costs in the potential for distraction from IVIS interactions on the road.

## Introduction

Operating a motor vehicle is one of the riskiest activities that adults engage in on a regular basis. In fact, roadway crashes are one of the leading causes of unintentional injury and death (World Health Organization [WHO], 2011; National Safety Council [NSC], 2017) and a significant percentage of crashes involve some form of distraction or inattention (e.g., Dingus et al., 2016). To safely operate a motor vehicle, drivers must maintain their eyes on the forward roadway and keep their mind focused on the drive. This becomes increasingly difficult with the prevalence of in-vehicle electronics. These systems change the way that drivers manage their attention behind the wheel, potentially leading to increases in driver distraction—especially as systems provide more information, functions, and features to drivers.

There are several components that factor into how distracting a secondary task is for the driver (e.g., Ranney et al., 2000; Regan et al., 2011; Strayer et al., 2011). One important factor is the cognitive demand associated with *Scanning, Predicting, Identifying, Deciding, and Executing Responses* (“SPIDER” – for a review see Strayer and Fisher, 2016). Performing cognitively demanding secondary tasks has been shown to impair each of these “SPIDER-related” processes and increase the relative risk of a crash (Fisher and Strayer, 2014). Another important factor is the visual demand associated with secondary-task interactions. Guidelines derived from the “radio tuning task” suggest that individual eye glances to a device while performing a secondary task should not exceed 2 s (Perez et al., 2013). Regardless of the type of secondary task, crash rates have been shown to systematically increase as the duration of glances away from the road increases (e.g., Simons-Morton et al., 2015). For example, when paired with the primary task of driving, texting is risky because it takes the driver’s eyes off the road for an average of 4.6 s (e.g., Drews et al., 2009; Olson et al., 2009). In many cases glances away from the forward roadway involve guiding a motor response (e.g., touching a location on the center stack screen). A final factor to consider is the duration of a distracting secondary task. Shutko and Tijerina (2006) suggest that task duration is critical because it represents the time in which an unexpected event might occur. All other things being equal, tasks that takes twice as long to complete will result in twice the potential risk of an adverse event.

Driver interactions with current and emerging in-vehicle information systems (IVIS) are often characterized by lengthy, complex, visual-manual, and auditory-vocal action sequences. For example, a driver may initiate a destination entry sequence with the press of a button on the steering wheel, followed by a verbal address entry, ending with the use of the touch screen. An earlier benchmarking effort from the Crash Avoidance Metrics Partnership (CAMP; Angell et al., 2006) evaluated a variety of older secondary tasks involving different types of visual, manual and cognitive interaction. Visual-manual tasks involved tuning the radio or adjusting fan speed using physical buttons located in the center console. Auditory-vocal tasks involved listening to an audiobook or sports broadcasts and answering related questions. This CAMP analysis found distinct profiles indicating that driver’s workload was multimodal and characterized by different combinations of visual, manual and cognitive components.

More recently, Strayer, Cooper, and colleagues reported on a program of research designed to understand the distraction potential associated with tasks now commonly available in new vehicles (Strayer et al., 2013; Strayer et al., 2014; Cooper et al., 2014; Strayer et al., 2017, 2018). This newer research examined some of the older task types evaluated with CAMP and also newer IVIS interactions that were not available in 2006. One important outcome of this research was a multimodal evaluation method for assessing the cognitive, visual, and temporal demands of complex multimodal IVIS interactions (see Strayer et al., 2017). Indeed, large variation in the distraction potential was observed with different tasks types (e.g., audio entertainment, calling and dialing, texting, and destination entry to support GPS navigation), and modes of interaction (e.g., center stack touchscreen; voice-commands).

Importantly, the reactions of older adults are often slower than those of younger adults, a phenomenon referred to as generalized slowing (e.g., Cerella, 1985; Salthouse, 1996). The age-related effects are magnified by the complexity of the interactions (Cerella et al., 1980). In fact, compared to differences in baseline reaction time (reflecting generalized slowing), the age-related differences more than doubled when participants used voice-based commands to select music or dial a phone number (Strayer et al., 2015). Because the duration of secondary task activities is greater for older than for younger drivers, age-related differences are expected to increase as the complexity of the secondary task increases. New in-vehicle systems and other secondary task activities may be especially problematic for older drivers (Albert et al., 2018). Ongoing research seeks to understand age-related differences in multitasking (Clapp et al., 2011) and the technology barriers that older drivers encounter (Vaportzis et al., 2017). However, little is known about the way in which drivers of any age interact with these complex multimodal In-Vehicle Information Systems (IVIS). These technologies have the potential to make driving safe and enjoyable. If they are not carefully implemented; however, they will decrease attention to the roadway.

Watson et al. (2011) suggested that the U-shaped function depicting crash rates and age is closely aligned with the maturation and decline in prefrontal cortical (PFC) regions of the brain (e.g., an inverted U-shaped function across the lifespan). The PFC regions are involved in a wide variety of higher-level cognitive/executive functions that support driving-related attention (e.g., scanning, predicting, identifying, deciding, and executing responses). In fact, laboratory studies have found greater multitasking costs for older adults (e.g., Craik, 1977; Hartley, 1992; Kramer and Larish, 1996; Hartley and Little, 1999; McDowd and Shaw, 2000) and Strayer et al. (2015) observed that older drivers experienced greater levels of cognitive demand with voice-based IVIS systems.

### Current Research

This study examined the cognitive and visual demand of younger and older drivers as they performed a variety of task types while driving a vehicle on a section of residential roadway. Workload measures were compared across two age groups and six different vehicles supporting different IVIS. The current research addressed two questions related to the use of these IVIS interactions.

Q1: Do the demands of IVIS interactions differ for older and younger drivers? If so, how?

Prior research has demonstrated that senescence is associated with declines in physical and cognitive performance that can impact safe driving. When older drivers interact with IVIS, they are more likely to experience cognitive, visual, or temporal interference. Furthermore, some types of IVIS interactions may present unique demands for older drivers.

Q2: Are some interfaces more difficult for older drivers to use? If so, why?

Research on IVIS voice interactions has found that older drivers experience higher cognitive demands when completing common tasks (e.g., Strayer et al., 2015). It is not clear, however, whether workload differences exist between older and younger drivers when completing tasks using controls housed in the center stack or when using center console controls. The ways in which older and younger drivers interact with IVIS may change the level of demand that they experience.

## Materials and Methods

### Participants

125 participants (52 females) were recruited via flyers, social media posts and local newsprint advertising with approval from the University of Utah Institutional Review Board (IRB). Eligible participants were native English speakers, had normal or corrected-to-normal vision, and held a valid driver’s license. Participants were also required to have proof of medical insurance and no accident involvement within the past 2 years. To ensure participants held a clean driving record, a Motor Vehicle Record report was obtained by the University of Utah’s Division of Risk Management.

All participants belonged to one of two age cohorts: younger drivers between 21 and 36 years of age (*M* = 24.8 years, St Dev = 2.97), and older drivers between 55 and 75 years of age (*M* = 65.8 years, St Dev = 5.36). Following University of Utah policy, participants were required to take and pass a 20-min online defensive driving course and certification test. Compensation was prorated at $20 per hour.

All participants were all healthy adult drivers with no physical or mental deficits. Younger and older participants self-reported health was 5.95 (0.85) vs. 6.10 (0.63) on a 7-point scale, a difference that was not statistically significant, *p* > 0.10. Younger and older participants drove an average 9.0 (7.19) vs. 8.8 (6.56) hours per week, a difference that was not statistically significant, *p* > 0.7. Younger and older participants reported an average of 7.38 (1.16) vs.7.38 (0.85) hours of sleep the night before testing, a difference that was not statistically significant, *p* > 0.98. Finally, no participants reported a history of neurological disorders.

Twenty-four individuals from each age cohort were tested in six unique vehicles, resulting in 48 participants per vehicle (i.e., each cell in the 6 × 2 factorial design had 24 participants). The study design allowed participants to drive all six vehicles, however this was not always possible. Participants were sample-matched by age and number of driving sessions in each of the evaluated vehicles; this was done to ensure that each age cohort was comprised of similar numbers of naive and repeat participants for the vehicle. The number of exposures were matched across vehicles and age cohorts as closely as possible; however, due to factors such as order of testing and availability of participants, exact matching was not possible. Thus, a planned missing data design was used (e.g., Graham et al., 2006; Little and Rhemtulla, 2013) as only eight individuals drove all six vehicles. Among the younger age cohort (21–36), 20 participants drove 1 vehicle, 16 drove 2 vehicles, 11 drove 3 vehicles, 7 drove 4 vehicles, 2 drove 5 vehicles, and 4 drove 6 vehicles. Among the older age cohort (55–75),: 28 participants drove 1 vehicle, 14 drove 2 vehicles, 10 drive 3 vehicles, 3 drove 4 vehicles, 6 drove 5 vehicles, and 4 drove 6 vehicles. Participants were initially naïve to the specific systems and tasks but were trained until they felt competent and confident performing each type of task while driving.

### Stimuli and Apparatus

#### Vehicles

The vehicles that were used for the study are listed below with the native infotainment system for each shown in parentheses. These cars were selected for inclusion in the study based on market diversity, availability, and IVIS functionality. Vehicles were acquired through Enterprise Rent-A-Car or purchased for testing.

• 2018 Audi A6 Premium (Man and Machine Intersect or MMI^®^)
• 2018 Cadillac CT6 Premium Luxury – Custom Packages (Custom User Experience or CUE^®^)
• 2018 Lincoln Navigator Select L (SYNC 3^®^)
• 2018 Mazda CX-5 Grand Touring (Mazda Connect^®^)
• 2018 Nissan Pathfinder SL (NissanConnect^®^)
• 2018 Volvo XC90 Momentum – Custom Packages (Sensus Connect^®^)

#### Equipment

Identical Google Pixel 2 phones on the T-Mobile network w Bluetooth-paired with each vehicle. An iPad Mini 4 (20.1 cm diagonal LED-backlit Multi-Touch display) was used to administer a visual-manual reference task (detailed below) and to survey participants on their self-reported measures of workload.

Each vehicle utilizes a variety of functions that facilitate interaction with the system such as touch screens, physical buttons, voice commands, touch/trackpads, and rotary wheels. Features were grouped into three Modes of Interaction: Voice Commands, Center Console, and Center Stack. IVIS functions were grouped into four Task Types: Audio Entertainment, Calling and Dialing, Text Messaging, and Navigation Entry.

Participants completed tasks involving interactions with the IVIS to achieve a goal (i.e., using the touch screen to tune the radio to a station, using voice commands to find a specified navigation destination, etc.) while driving. Tasks were categorized into four Task Types and three Modes of Interaction.

The possible Task Types performed by the participant were:

• Audio Entertainment: Participants tuned the radio to specific AM and FM frequencies and selected music from a USB connected iPad mini, using designated categories such as song titles, music genres, artist names, and album titles.
• Calling and Dialing: A list of 91 contacts with a mobile and/or work number was created for participant testing. Participants were instructed to call designated contacts and the associated number type was specified when applicable. In vehicles capable of dialing phone numbers, participants were instructed to dial the phone number 801-555-1234 as well as their own phone number.
• Text Messaging: Participants were provided with hypothetical scenarios in which they received text messages from various contacts and were instructed to interact with the messages using specified Modes of Interaction. Vehicles varied in their SMS capabilities. A portion of the system/Mode of Interaction combinations allowed users to listen to messages and reply with predetermined responses, or solely listen to the messages and not respond. Other vehicles and Modes of Interaction allowed users to respond to text messages using free dictation.
• Navigation Entry: Participants started and canceled route guidance to different locations based on a hypothetical situation they were given that differed slightly according to the options available in each system.

The Modes of Interaction with each system are described below. Interaction modalities were selected based on compatibility with the specific tasks.

• Center Stack: Visual-manual tasks were performed using the center stack interfaces found in the middle of the dash to the right of the driver. Center stack systems generally include a touchscreen to integrate visual/manual interactions so that drivers can select options and navigate menus via touch, scroll bars, seek arrows, etc. to complete tasks using options displayed on the screen. Some vehicles provide physical buttons near the touch screen for selection of options.
• Center Console: Vehicles utilizing center console controls replace or augment a touchscreen interface or manual center stack controls with an interface usually consisting of a rotary wheel and manual buttons in the center console to the right of the driver. The center console controls facilitate interactions with the center stack display located in the middle of the dash. The rotary can be spun to scroll through menus and used like a button to make selections. In some cases, the rotary wheel interfaces can be maneuvered in various directions to navigate menus, like a joystick. In the case of the Audi A6, the center console controls also incorporated a touch-sensitive pad that could be used to draw letters and numbers in search functions or select preset radio stations.
• Auditory Vocal: The voice-based interaction with each IVIS system is initiated by the press of a physical voice recognition button on the steering wheel. Microphones installed in the vehicle process the driver’s verbal commands and assist them while performing tasks in the vehicle. Possible voice command options may be presented audibly or displayed on the vehicle’s center stack or instrument cluster to assist users in achieving their goal.

The configuration of Task Types and Modes of Interaction depended on each system’s unique capabilities. All vehicles supported Voice Commands, however each vehicle differed on visual/manual interaction (e.g., touchscreen, manual buttons, center console controls). Furthermore, different systems required specific syntax or commands to be given in a systematic order to accomplish tasks in different interaction modes. Task lists were developed to test the various combinations of features and functions available in each system. Tasks were standardized across systems as much as possible, given the variability in system interactions. The tasks supported for each vehicle system are noted in Table 1. A complete list of all task instructions for each vehicle is provided in Supplementary Appendix A.

**TABLE 1:** Modes of Interaction and tasks types for the 6 different vehicles.

**Vehicle**	**Audi**		**Cadillac**		**Lincoln**		**Mazda**		**Nissan**		**Volvo**	
	**A6**		**CT6**		**Navigator**		**CX-5**		**Pathfinder**		**XC90**	
**Mode of interaction**	**CS**	**VC**	**CC**	**VC**	**CS**	**VC**	**CC**	**VC**	**CS**	**VC**	**CS**	**VC**
Audio entertainment	✓	✓	✓	✓	✓	✓	✓	✓	✓	✓	✓	✓
Calling and dialing	✓	✓	✓	✓	✓	✓	✓	✓	✓*	✓	✓	✓
Navigation	✓	✓	✓	✓	✓*	✓	✓**	✓**	✓***	✓	✓	✓
Text messaging	✓	✓	✓	–	✓*	✓**	✓	✓	✓	✓	–	✓

*Mode of Interaction: CS = Center Stack; VC = Voice Commands, CC = Center Console. Cells with checks indicate the availability of tasks for that Vehicle/Mode of interaction. Asterisks indicate cases where a variation in task instructions was required to accommodate vehicle capabilities or limitations. Empty cells represent tasks that were not available for that Vehicle/Mode of Interaction (e.g., The Cadillac CT6 did not provide Text Messaging functionality through voice controls). The full task instruction set for each Vehicle/Mode of Interaction is provided in Supplementary Appendix A.*

#### Detection Response Task

Participants were trained to respond to both a vibrotactile stimulus and a remote visual stimulus (cf. International Organization for Standardization, 2015). A vibrotactile stimulus was positioned under the participant’s left collarbone, and a remote LED light was placed along a strip of Velcro on the dashboard in such a manner that the participants only saw the reflection of the light as it changed from orange to red in the windshield directly in the forward line of sight (see Castro et al., 2016; Cooper et al., 2016). This variant of the standard DRT was used to maximize sensitivity to both cognitive and visual attention. Reaction time to the vibrotactile stimulus was used to assess cognitive workload while hit rate to the forward LED was used as a measure of competing visual demand.

A microswitch (i.e., small button) was attached to either the index or middle finger of the left hand and pressed against the steering wheel by participants when they felt a vibration or saw the light change colors. Each press of the switch was counted and recorded but only the first response was used to determine response time to the stimulus. Response time with sub-millisecond resolution to the vibrotactile onset or LED light was recorded using a dedicated microprocessor that passed results over USB connection to the host computer for storage and later analysis.

Following International Organization for Standardization (2015), the vibrotactile device emitted a small vibration stimulus, like a vibrating cell phone. The remote light stimulus consisted of a change in color from orange to red. This color changing LED stimulus differed from the ISO standard (see Castro et al., 2016). The occurrences of these stimuli cued the participant to respond as quickly as possible by pressing the microswitch against the steering wheel. The tactile and light stimuli were equiprobable and were programed to occur every three to 5 s (with a rectangular distribution of inter-stimulus intervals within that range) and lasted for 1 s or until the participant pressed the microswitch.

### Procedure

#### Driving Route

**FIGURE 1:**
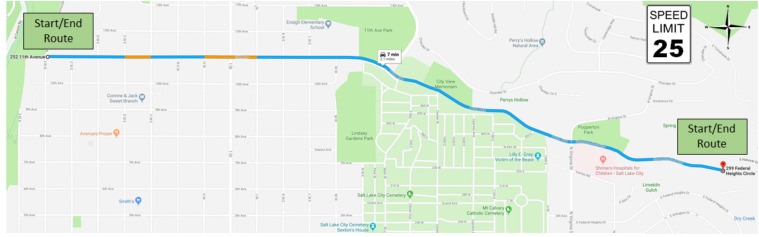
A map image of the designated driving route, a two-mile-long residential roadway in Salt Lake City, Utah.

A suburban residential street with a 25-mph speed limit was used for the on-road driving study. The route was a straight road with four stop signs and two speed bumps. Participants were required to follow all traffic laws and adhere to the 25-mph speed limit. The driving route was approximately two miles long one-way with an average drive time of 6 min. A researcher was present in the passenger seat of each vehicle for safety monitoring and data collection. An image of the driving route can be seen in Figure 1. The respective start/end points of the driving course were 40.781944, −111.8820912 and 40.7770036, −111.8438273.

#### Training

Prior to the start of the study, participants were provided the time to become accustomed to the vehicle, the route, and the DRT. The initial familiarization period is as follows:

• DRT training: Participants were instructed on how to respond to the light and vibration motor using the microswitch. Researchers monitored participants as they practiced responding to 10 stimuli to ensure participants produced response times of less than 500 milliseconds, indicating a competence and understanding of the task. After initial training, participants were given the opportunity to practice responding to the DRT while driving during the practice drive described below.
• Practice route: Prior to data collection, participants were instructed to drive the route seen in Figure 1. Researchers pointed out all obvious and identifiable road hazards. This practice drive allowed participants to familiarize themselves with the road as well as the handling of the vehicles.

Participants were trained to interact with and complete tasks using the assigned Mode of Interaction before each condition began. Participants were required to complete three task trials without error, while simultaneously responding to the DRT prior to starting the driving task for each of the system interactions.

#### Experimental Blocks

During the experimental blocks, participants were instructed to complete a set of tasks administered by the researcher using an assigned Mode of Interaction with the infotainment system. Driving the vehicle was emphasized as task prioritization and was expressed to participants in verbal instructions.

Participants were asked to pull over on the side of the road at the termination of one length of the route. The subsequent experimental block, equipped with a new assigned Task Type and Mode of Interaction, began in the opposite direction on the same route and concluded in the same manner. This was repeated until all conditions were completed, resulting in alternating travel directions for each experimental block. The order of Task Types and conditions administered in each vehicle was counterbalanced across 24 participants in each Age Cohort.

Tasks were only administered in safe and normal driving conditions. Disruptions to the natural driving environment resulted in the researcher instructing the participant to terminate the current task and only administering a new task when it was safe to do so. Tasks were not administered as participants approached intersections or construction zones. Normal behaviors of other vehicles and pedestrians were within the scope of the natural driving environment.

Participants were provided with verbal hypothetical situations or commands as cues from the passenger researcher (e.g., “Jack Olsen would like you to call him on his cell phone.”). Participants were trained to wait to start each task until the researcher said “go.” After the completion of each task, participants were trained to say, “done.” Tasks were delivered with an approximately 5 s interval between the participants’ announcement of completion and the researchers’ administration of the next task. Researchers denoted each task’s start and end time by pressing designated keys on the data collection computer, thus indicating the timing of on-task performance on the driving route. DRT trials were considered valid for inclusion in the statistical analysis if they occurred between these start and end times. Participants were encouraged to complete tasks as efficiently as possible, however drivers were given as much time as needed to complete each task, unless the end of the route was reached in which case tasks were terminated prematurely and later omitted from analysis.

Participants also performed three control tasks while driving one length of the designated route per task. These tasks provided a standard set of performance references which included a single-task baseline, a high cognitive demand reference task (nBack), and a high visual-demand reference task (SuRT).

• Single-task Baseline: Participants performed a single-task baseline drive using the vehicle being tested on the designated route, without interacting with the infotainment system. During the single-task baseline, participants interacted solely with the DRT stimuli, responding to both the tactile stimulus and light change as fast as possible and were asked to remain silent as to minimize distraction.
• Auditory nBack task: The auditory nBack task (Mehler et al., 2011) presented a pre-recorded, randomized set of numbers ranging from zero to nine in sequences of 10. In each sequence, numbers were spoken aloud at a rate of one digit every 2.25 s. Participants were instructed to verbally repeat the number that was presented two trials earlier as they concurrently listened for the next number in the sequence. Participants were told to respond as accurately as possible to the nBack stimuli while researchers monitored performance in real-time. During the nBack task, participants also responded to the DRT stimuli.
• Surrogate Reference Task (SuRT): A modernized version of the SuRT task (International Organization for Standardization, 2012) was presented on an iPad Mini 4 with circles printed in black on a white background. A target was presented on the display amidst 21–27 distractors. The target was an open circle 1.5 cm in diameter and the distractors were open circles 1.2 cm in diameter. Participants’ were instructed to touch the location of the target. Immediately thereafter, a new display was presented with a different configuration of targets and distractors. The location of targets and distractors was randomized across the trials in the SuRT task. Participants were instructed to continuously perform the SuRT task while giving the driving task highest priority as researchers monitored performance in real-time. Researchers instructed participants to pause the SuRT task at intersections and in the event of potential hazards on the roadway. During the SuRT task, participants also responded to the DRT stimuli.

After the completion of each condition, participants completed the NASA-TLX (Hart and Staveland, 1988) to assess the subjective workload of the system presented on the iPad Mini 4. Following this assessment, participants were asked an open-ended question as an opportunity to describe or detail information not captured by the restrictive NASA-TLX questions: “Do you have any comments about the task or vehicle after this last run?”

### Dependent Measures

Detection Response Task data were preprocessed following procedures outlined in International Organization for Standardization (2015). All response times faster than 100 milliseconds which were considered impossible or inadvertent responses and were not considered valid. Similarly, reaction times slower than 2500 milliseconds were eliminated from our overall calculation for Reaction Time. Non-responses or responses that were made after 2500 milliseconds from the stimulus onset were coded as a miss. System interaction was recorded by the researcher via pressing designated keys on the DRT host computer, allowing the identification of “on-task” and “off-task” segments of driving. Incomplete, interrupted, or otherwise invalid tasks, were marked with a key-flag and excluded from the analysis. The dependent measures obtained in the study are listed below:

• DRT – Reaction Time: Defined as the sum of all valid reaction times to the DRT task divided by the number of valid reaction times. Reaction time to the DRT was used to calculate cognitive demand during each experimental condition. This was used to gauge the approximate mental workload and allocation of cognitive resources required by the task for each type of IVIS interaction. Reaction times to both stimuli were included in analysis.
• DRT – Hit Rate: Defined as the number of valid responses divided by the total number of valid stimuli presented during each condition. Hit Rate to the DRT was used to calculate visual demand, or how much visual attention was required by the task during each experimental condition. In order to maximize the sensitivity to divided visual attention effects (e.g., looking away from the forward roadway), analyses were only conducted on responses to the remote LED stimulus.

Task Completion Time was obtained from the time stamp on the DRT data file defined as the time researchers said “go” and participants first initiated an action to the time when that action was completed, and the participant said, “done.” Tasks with irregular occurrences and errors in administration or performance that may have affected Task Completion Time were marked as abnormal during data collection and were not included in subsequent analyses. When assessed using the visual occlusion methodology, the NHTSA guidelines provide an implicit upper limit of 24 s of total task time (National Highway Transportation Safety Administration [NHTSA], 2013). While originally intended for visual/manual tasks, these guidelines provide a reasonable upper limit for task durations of any type.

### Experimental Design

The experimental design was a 2 (Age Cohort: older or younger drivers) x 4 (Task Type: Audio Entertainment, Calling and Dialing, Navigation Entry, and Text Messaging) x 3 (Mode of Interaction: Auditory/Vocal, Center Stack, Center Console) factorial with 24 participants evaluated in each cell of the factorial. However, not all system interactions offered the full factorial design (i.e., not all Task Types and Modes of Interaction were available in all vehicles).

### Data Analysis

Linear mixed effects analyses were performed using R 3.5.1 (R Core Team, 2018) and lme4 version 1.1-18-1 (Bates et al., 2015). In the analyses reported below, models containing the Age Cohort plus one additional factor (i.e., Task Type and Mode of Interaction) were compared to a model without the effect in question. All main effects and two-way interactions with Age Cohort were analyzed and are included below. *P*-values were obtained by likelihood ratio tests comparing the full linear mixed effects model to the partial linear mixed effects model (see Winter, 2013). This linear mixed modeling analysis has the advantage of analyzing all available data while adjusting fixed effect, random effect, and likelihood ratio test estimates for missing data. The full analysis script is available for download here: https://github.com/utahcdst/Aging-Report-Frontiers.

Pairwise comparisons for each of the analyses are provided in a tabular format see Tables 2–9. Pairwise comparisons were extracted through the sequential evaluation of each model in question using a factor re-referencing approach. Each table of pairwise comparisons is structured to provide the (1) the means and standard deviations at each factor level by age, (2) the pairwise comparisons for the age contrast, which indicates whether the effect of age was significant at each level of the factor in question, (3) the pairwise comparisons of the effect in questions, which indicates whether factor levels differed from each other, and (4), the pairwise comparisons for the effect of age at each level of the factor in question. This indicates whether the age effects at each factor level differed from each other. This selective set of pairwise comparisons addresses the core effects of interest.

**TABLE 2:** Pairwise comparisons for task completion time as a function of task type and age cohort.

	**Task completion time by task type**	**Audio entertainment**	**Calling and dialing**	**Text messaging**	**Navigation entry**
Means and SD	Ages 21–36 (*n* = 24) Mean (SD)	18.0 (5.01)	17.7 (3.64)	27.7 (11.8)	31.4 (8.63)
	Ages 55–75 (*n* = 24) Mean (SD)	25.4 (10.7)	22.4 (6.48)	33.8 (20.4)	40.0 (14.1)
Age cohort contrasts: *t*-value		7.00**	4.64**	5.53**	8.00**
Task type contrasts: *t*-value	Audio entertainment				
	Calling and dialing	−2.86*			
	Text messaging	14.53**	17.25**		
	Navigation entry	23.86**	26.71**	8.20**	
Task type contrasts by	Audio entertainment				
age cohort: *t*-value	Calling and dialing	–2.35			
	Text messaging	–1.11	1.12		
	Navigation entry	1.02	3.36**	2.08	

*p < 0.01, **p < 0.001.

**TABLE 3:** Pairwise coparisons for task completion time as a function of mode of interaction and age cohort.

	**Mode of interaction**	**Auditory vocal**	**Center console**	**Center stack**
Means and SD	Ages 21–36 (*n* = 24) Mean (SD)	26.2 (11.3)	23.2 (7.85)	19.7 (6.42)
	Ages 55–75 (*n* = 24) Mean (SD)	32.7 (17.2)	31.4 (13.3)	25.7 (11.3)
Age cohort contrasts		6.98**	5.93**	6.01**
Mode of interaction contrasts	Auditory vocal			
	Center console	−4.10**		
	Center stack	−11.03**	−4.36**	
Mode of interaction contrasts by age cohort	Auditory vocal			
	Center console	–0.95		
	Center stack	–0.98	–0.98	

*p<0.01, **p<0.001.

**TABLE 4:** Pairwise comparisons for reaction time as a function of task type and age cohort.

	**Task type**	**Single**	**Audio entertainment**	**Calling and dialing**	**Text messaging**	**Navigation entry**	**nBack**	**SuRT**
Means and SD	Ages 21–36 (*n* = 24) Mean (SD)	526 (110)	778 (154)	762 (150)	763 (158)	773 (157)	760 (174)	703 (147)
	Ages 55–75 (*n* = 24) Mean (SD)	639 (135)	946 (184)	934 (166)	951 (176)	954 (175)	944 (151)	881 (182)
Age cohort contrasts		4.50**	7.98**	7.34**	7.91**	7.70**	7.47**	7.16**
Task type contrasts	Single							
	Audio entertainment	38.56**						
	Calling and dialing	35.34**	−3.94**					
	Text messaging	35.25**	–2.46	1.28				
	Navigation entry	37.49**	–1.27	2.66*	1.25			
	nBack	31.17**	–2.57	0.65	–0.44	–1.53		
	SuRT	24.33**	−10.53**	−7.31**	−8.14**	−9.48**	−6.89**	
Task type contrasts	Single							
by age cohort	Audio entertainment	4.99**						
	Calling and dialing	3.99**	–1.22					
	Text messaging	4.90**	0.10	1.26				
	Navigation entry	4.56**	–0.52	0.70	–0.59			
	nBack	4.31**	–0.01	0.99	–0.09	0.42		
	SuRT	3.85**	–0.55	0.45	–0.61	–0.12	–0.46	

*p<0.01, **p<0.001.

**TABLE 5:** Pairwise comparisons for reaction time as a function of mode of interaction and age cohort.

	**Mode of interaction**	**Single**	**Auditory vocal**	**Center console**	**Center stack**	**nBack**	**SuRT**
Means and SD	Ages 21–36 (*n* = 24) Mean (SD)	526 (110)	764 (161)	775 (147)	775 (148)	760 (174)	703 (147)
	Ages 55–75 (*n* = 24) Mean (SD)	639 (135)	954 (188)	957 (160)	942 (164)	944 (151)	881 (182)
Age cohort contrasts:		4.49**	8.37**	7.34**	7.35**	7.47**	7.16**
Mode of interaction contrasts	Single						
	Auditory vocal	40.08**					
	Center console	34.28**	0.89				
	Center stack	37.84**	0.04	–0.78			
	nBack	31.09**	–0.94	–1.46	–0.91		
	SuRT	24.27**	−9.60**	−8.72**	−9.08**	−6.87**	
Mode of interaction contrasts	Single						
by age cohort	Auditory vocal	5.63**					
	Center console	4.05**	–0.88				
	Center stack	3.98**	–1.92	–0.61			
	nBack	4.30**	–0.22	0.49	1.13		
	SuRT	3.84**	–0.80	0.00	0.58	–0.46	

*p<0.01, **p<0.001.

**TABLE 6:** Pairwise comparisons for hit rate as a function of task type and age cohort.

	**Task Type**	**Single**	**Audio entertainment**	**Calling and dialing**	**Text messaging**	**Navigation entry**	**nBack**	**SuRT**
Means and SD	Ages 21–36 (*n* = 24) Mean (SD)	0.90 (0.11)	0.59 (0.24)	0.66 (0.24)	0.66 (0.21)	0.60 (0.24)	0.76 (0.19)	0.65 (0.18)
	Ages 55–75 (*n* = 24) Mean (SD)	0.80 (0.20)	0.29 (0.24)	0.37 (0.26)	0.35 (0.23)	0.33 (0.23)	0.53 (0.25)	0.44 (0.26)
Age cohort contrasts		−3.79**	−10.34**	−9.78**	−10.01**	−9.29**	−7.26**	−6.67**
Task type contrasts	Single							
	Audio entertainment	−34.73**						
	Calling and dialing	−28.55**	7.56**					
	Text messaging	−28.47**	6.18**	–1.02				
	Navigation entry	−32.37**	2.85*	−4.69**	−3.45**			
	nBack	−15.12**	17.27**	11.10**	11.58**	14.92**		
	SuRT	−22.17**	9.19**	3.01*	3.76**	6.85**	−7.02**	
Task type contrasts by	Single							
age cohort	Audio entertainment	−8.69**						
	Calling and dialing	−7.92**	0.94					
	Text messaging	−8.22**	0.22	–0.67				
	Navigation entry	−7.24**	1.76	0.83	1.46			
	nBack	−4.55**	3.43**	2.66*	3.13**	1.99		
	SuRT	−3.77**	4.35**	3.58**	4.02**	2.90*	0.79	

*p<0.01, **p<0.001.

**TABLE 7:** Pairwise comparisons for hit rate as a function of mode of interaction and age cohort.

	**Mode of interaction**	**Single**	**Auditory vocal**	**Center console**	**Center stack**	**nBack**	**SuRT**
Means and SD	Ages 21–36 (*n* = 24) Mean (SD)	0.90 (0.11)	0.70 (0.22)	0.54 (0.23)	0.56 (0.23)	0.76 (0.19)	0.65 (0.18)
	Ages 55–75 (*n* = 24) Mean (SD)	0.80 (0.20)	0.40 (0.25)	0.21 (0.18)	0.30 (0.22)	0.53 (0.25)	0.44 (0.26)
Age cohort contrasts:		−3.85**	−10.53**	−10.26**	−9.42**	−7.38**	−6.77**
Mode of interaction	Single						
contrasts	Auditory vocal	−29.47**					
	Center console	−37.62**	−16.84**				
	Center stack	−39.48**	−16.84**	3.02*			
	nBack	−16.00**	9.32**	20.72**	20.47**		
	SuRT	−23.46**	–0.01	12.92**	11.68**	−7.42**	
Mode of interaction	Single						
contrasts by age cohort	Auditory Vocal	−9.61**					
	Center console	−8.82**	–0.99				
	Center stack	−7.80**	1.84	2.25			
	nBack	−4.83**	3.53**	3.71**	2.05		
	SuRT	−4.00**	4.60**	4.61**	3.06*	0.85	

*p<0.01, **p<0.001.

**TABLE 8:** Pairwise comparisons for NASA-TLX subjective responses as a function of task type and age cohort.

	**Task type**	**Single**	**Audio entertainment**	**Calling and dialing**	**Text messaging**	**Navigation entry**	**nBack**	**SuRT**
Means and SD	Ages 21–36 (*n* = 24) Mean (SD)	3.75 (2.65)	8.23 (4.33)	7.04 (4.15)	7.26 (3.88)	8.45 (4.32)	11.4 (3.73)	10.4 (4.25)
	Ages 55–75 (*n* = 24) Mean (SD)	5.08 (3.27)	10.3 (4.79)	8.03 (4.53)	8.68 (4.23)	10.0 (4.54)	12.1 (4.09)	10.1 (4.53)
Age cohort contrasts		2.51	4.29**	2.15	2.85*	3.16*	1.50	–0.11
Task type contrasts	Single							
	Audio entertainment	20.17**						
	Calling and dialing	12.93**	−8.86**					
	Text messaging	14.24**	−6.80**	1.85				
	Navigation entry	19.88**	–0.37	8.50**	6.45**			
	nBack	26.28**	10.19**	17.42**	15.61**	10.50**		
	SuRT	20.95**	4.01**	11.25**	9.54**	4.31**	−5.37**	
Task type contrasts by	Single							
age cohort	Audio entertainment	1.65						
	Calling and dialing	–0.75	–2.93					
	Text messaging	0.08	–1.87	0.98				
	Navigation entry	0.38	–1.56	1.37	0.36			
	nBack	–1.10	–2.91	–0.53	–1.32	–1.64		
	SuRT	−2.86*	−4.95**	–2.56	–3.32	–3.68	–1.75	

*p<0.01, **p<0.001.

**TABLE 9:** Pairwise comparisons for NASA-TLX subjective responses as a function of mode of interaction and age cohort.

	**Mode of interaction**	**Single**	**Auditory vocal**	**Center console**	**Center stack**	**nBack**	**SuRT**
Means and SD	Ages 21–36 (*n* = 24) Mean (SD)	3.75 (2.65)	6.57 (3.79)	9.02 (4.24)	8.98 (4.33)	11.4 (3.73)	10.4 (4.25)
	Ages 55–75 (*n* = 24) Mean (SD)	5.08 (3.27)	8.12 (4.39)	10.4 (4.2)	10.6 (4.74)	12.1 (4.09)	10.1 (4.53)
Age cohort contrasts		2.55	3.42	2.93	3.21	1.53	–0.10
Mode of interaction contrasts	Single						
	Auditory vocal	13.60**					
	Center console	20.29**	11.70**				
	Center stack	23.46**	15.56**	0.69			
	nBack	27.00**	20.54**	8.29**	8.66**		
	SuRT	21.52**	13.61**	2.46	2.12	−5.52**	
Mode of interaction contrasts	Single						
by age cohort	Auditory vocal	0.43					
	Center console	0.29	–0.09				
	Center stack	0.34	–0.10	0.01			
	nBack	–1.13	–1.86	–1.49	–1.68		
	SuRT	−2.93*	−4.14**	−3.40**	−3.82**	–1.80	

*p<0.01, **p<0.001.

For each independent variable (Task Completion Time, DRT Reaction Time, DRT Hit Rate, Subjective Workload), Linear Mixed Effects Models were built to explore the main effects of Age Cohort, Task Type, and Mode of Interaction as well as all 2-way interactions with Age Cohort (e.g., Age Cohort by Task Type and Age Cohort by Mode of Interaction).

Where appropriate, results were analyzed and modeled with the inclusion of the baseline tasks (Single-task, SuRT, nBack). Baseline tasks were not included in the analysis of Task Completion Time. Results address the question of whether there were significant age differences in the associations of interactions with the vehicle technology with the independent variables.

Mean results for each of the main effects are provided in the units in which they were recorded, along with the standard error (SE) in parentheses. Due to the number of statistical comparisons performed, we used a more conservative α = 0.01 and α = 0.001 to denote varying levels of statistical significance. Effects that reach these levels are flagged with a single ‘^∗^‘ and a double ‘^∗∗^‘ respectively. This more conservative significance level helps to reduce the likelihood of false positives in the statistical analysis.

## Results

### Task Completion Time

#### Main Effects

Results indicated that Task Completion Time differed by Age Cohort, χ^2^(1) = 51.42, *p* < 0.001 (Younger: *M* = 23.5, *SD* = 9.83; Older: *M* = 30.2, *SD* = 15.2) with older drivers taking significantly longer to complete tasks than younger drivers. Additionally, there were significant main effects of Task Completion Time for Task Type, χ^2^(3) = 785.85, *p* < 0.001 (Audio Entertainment: *M* = 21.6, *SD* = 9.09; Calling and Dialing: *M* = 20.0, *SD* = 5.76; Text Messaging: *M* = 30.7, *SD* = 35.6; Navigation Entry: *M* = 30.2, *SD* = 15.2), and Mode of Interaction, χ^2^(2) = 119.56, *p* < 0.001 (Auditory Vocal: *M* = 29.4, *SD* = 14.9; Center Console: *M* = 27.4, *SD* = 11.7; Center Stack: *M* = 22.6, *SD* = 9.64).

#### Age Cohort by Task Type

The analysis revealed a significant two-way interaction between Age Cohort and Task Type, χ^2^(1, 3) = 12.52, *p* = 0.006. Age contrasts indicated that the effects of age reached significance at all levels of Task Type and that all levels of Task Type differed from each other. Furthermore, Task Type contrasts by Age Cohort suggests that older drivers had an especially difficult time with the Navigation Entry task. Notably, only the median Task Completion Time for the Audio Entertainment and Calling and Dialing tasks came in under the 24-s referent for both age groups (see Strayer et al., 2013).

#### Age Cohort by Mode of Interaction

The interaction between Mode of Interaction and Age Cohort was not significant, χ^2^(1, 2) = 1.09, *p* = 0.57, suggesting that the increased Task Completion Time for older drivers was similar across all three Modes of Interaction. That is, while Age Cohort and Mode of Interaction both affected Task Completion Time, the impact of each was not dependent on the other. Age contrasts indicated that the effect of age reached significance for all levels of Mode of Interaction. Mode of Interaction contrasts indicated that performance in each Mode of Interaction differed between performance in the other Modes of Interaction. Mode of Interaction contrasts by Age Cohort suggested that the magnitude of the Cohort effect was not dependent on the specific.

#### Age Cohort by Mode of Interaction by Task Type

**FIGURE 2:**
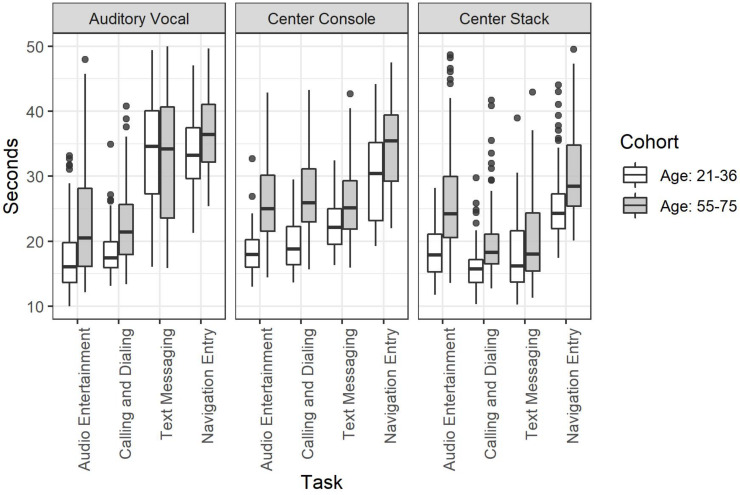
Task completion time for the full factorial of age cohort by mode of interaction by task.

The three-way interaction between each of these factors was significant, χ^2^(1, 3, 2) = 24.8, *p* < 0.001. This higher order interaction suggests that the effect of Age was dependent on the specific Task/Mode combination Figure 2.

### DRT Reaction Time

#### Main Effects

Results indicated that DRT Reaction Time differed by Age Cohort, χ^2^(1) = 49.8, *p* < 0.001 (Young: *M* = 739, *SD* = 168; Older: *M* = 914, *SD* = 193), with older drivers taking significantly longer, on average, to respond to the DRT than younger drivers. Additionally, there were significant main effects for Task Type, χ^2^(6) = 1466, *p* < 0.001 (Audio Entertainment: *M* = 870, *SD* = 194; Calling and Dialing: *M* = 848, *SD* = 180; Text Messaging: *M* = 856, *SD* = 191; Navigation Entry: *M* = 863, *SD* = 189), and Mode of Interaction, χ^2^(5) = 1450, *p* < 0.001 (Auditory Vocal: *M* = 858, *SD* = 199; Center Console: *M* = 868, *SD* = 179; Center Stack: *M* = 856, *SD* = 177).

#### Age Cohort by Task Type

Analysis of the two-way interaction between Age Cohort and Task Type indicated that the interaction reached significance, χ^2^(1, 6) = 31.6, *p* < 0.001. Inspection of the data reveals a highly consistent effect of task engagement and age across each of the Task Types. Age Cohort contrasts indicated that the effect of age reached significance at each level of Task Type. Task Type contrasts suggest that Reaction Time to the Audio Entertainment task was slightly more delayed than to the Calling and Dialing task and that Reaction Time between the Calling and Dialing and Navigation Entry tasks also reached significance. Reaction time to the Single Task baseline was faster than to all other Task Types. Reaction time to the SuRT task was also faster than to the other Task Type (except Single Task), while Reaction Time to the nBack task did not differ from the Reaction Times to the four IVIS Task Types. Task Type contrasts by Age Cohort suggest that the effect of Age Cohort was smallest for the Single Task condition but that it did not differ between any of the other conditions.

#### Age Cohort by Mode of Interaction

The interaction between Age Cohort and Mode of Interaction also reached significance, χ^2^(1, 2) = 33.05, *p* > 0.001. Age Cohort contrasts indicated that the effect of Age Cohort on Reaction Time reached significance for each level of the Mode of Interaction. Mode of Interaction contrasts indicated that the Single Task and SuRT tasks differed from all other Modes of Interaction but that none of the other Modes of Interaction differed from each other. Mode of Interaction contrasts by Age Cohort indicated that the effect of Age Cohort was smallest in the Single Task baseline and similar in all other conditions.

#### Age Cohort by Mode of Interaction by Task Type

**FIGURE 3:**
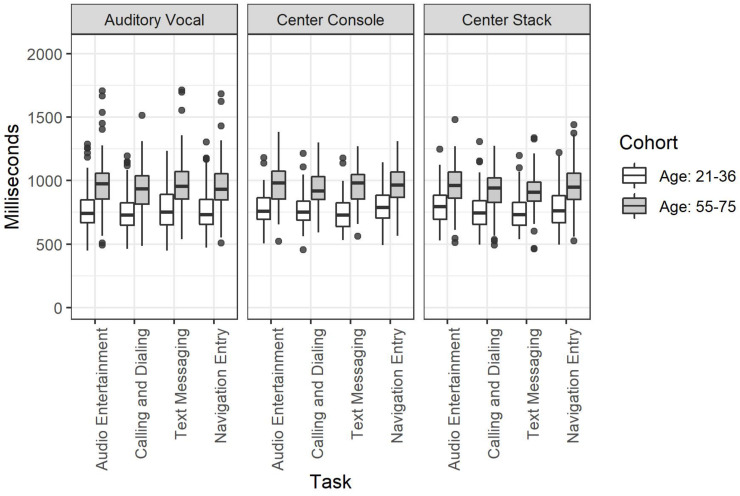
Detection response task reaction time for the full factorial of age cohort by mode of interaction by task.

The three-way interaction between each of these factors was not significant, χ^2^(1, 3, 2) = 1.69, *p* = 0.946. This lack of interaction is clearly visible in Figure 3 where a main effect of age is apparent with highly consistent effects of Mode of Interaction and Task Type.

### DRT Hit Rate

#### Main Effects

Results indicated that Hit Rate differed by Age Cohort, χ^2^(1) = 70.2, *p* < 0.001 (Young: *M* = 0.67, *SD* = 0.23; Older: *M* = 0.41, *SD* = 0.28), with younger drivers detecting and accurately responding to the onset of the forward LED significantly more often than older drivers. Consistently across factors, older drivers failed to respond to the light stimulus more frequently resulting in lower Hit Rates. Additionally, there was a significant main effect of Task Type, χ^2^(6) = 1232, *p* < 0.001 (Audio Entertainment: *M* = 0.44, *SD* = 0.28; Calling and Dialing: *M* = 0.51, *SD* = 0.29; Text Messaging: *M* = 0.50, *SD* = 0.27; Navigation Entry: *M* = 0.47, *SD* = 0.27), and Mode of Interaction, χ^2^(5) = 1577, *p* < 0.001 (Auditory Vocal: *M* = 0.55, *SD* = 0.28; Center Console: *M* = 0.38, *SD* = 0.26; Center Stack: *M* = 0.43, *SD* = 0.26).

#### Age Cohort by Mode of Interaction

The interaction between Age Cohort and Mode of Interaction was also significant, χ^2^(1, 5) = 116, *p* > 0.01, suggesting that the effect of age cohort on Hit Rate depended on the Mode of Interaction. Age contrasts indicated that the effect of Age Cohort reached significance at all levels of the Mode of Interaction. Mode contrasts suggest that Hit Rates were highest in the Single Task condition, followed by the nBack task, then the SuRT and Auditory Vocal tasks, and lowest in the Center Stack and Center Console Modes of Interaction. Mode of Interaction contrasts by Age Cohort suggest that the effect of Age Cohort was similarly large for each of the Modes of Interaction, somewhat smaller in the nBack and SuRT tasks, and smallest in the Single Task.

#### Age Cohort by Mode of Interaction by Task Type

**FIGURE 4:**
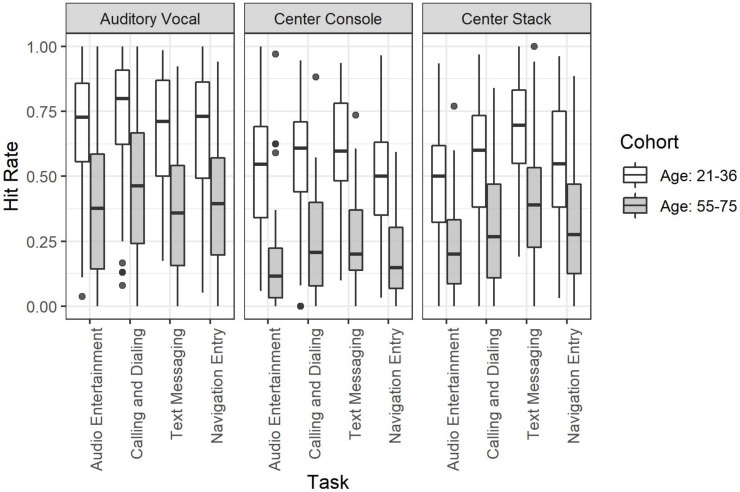
Detection response task hit rate for the full factorial of age cohort by mode of interaction by task.

The three-way interaction between each of these factors was not significant, χ^2^(1, 3, 2) = 1.33, *p* = 0.969. This lack of interaction is visible in Figure 4 where a main effect of age is apparent with highly consistent effects of Mode of Interaction and Task Type.

### Subjective Workload

#### Main Effects

Results indicated that the composite TLX scores (average of the 21-point rating across each of the TLX subscales) differed by Age Cohort, χ^2^(1) = 8.69, *p* = 0.003 (Young: *M* = 7.96, *SD* = 4.44; Older: *M* = 9.23, *SD* = 4.72), with older drivers reporting IVIS task interactions to be more difficult than younger drivers. Additionally, there were significant main effects for Task Type, χ^2^(6) = 789, *p* < 0.001 (Audio Entertainment: *M* = 9.27, *SD* = 4.68; Calling and Dialing: *M* = 7.53, *SD* = 4.37; Text Messaging: *M* = 7.96, *SD* = 4.12; Navigation Entry: *M* = 9.22, *SD* = 4.49), and Mode of Interaction, χ^2^(5) = 958, *p* < 0.001 (Auditory Vocal: *M* = 7.33, *SD* = 4.17; Center Console: *M* = 9.70, *SD* = 4.27; Center Stack: *M* = 9.75, *SD* = 4.60).

#### Age Cohort by Task Type

Analysis of the two-way interaction between Age Cohort and Task Type on Subjective Workload indicated that the interaction was not significant, χ^2^(1, 6) = 28.4, *p* > 0.001. Age Cohort Contrasts suggest that the effect of Age Cohort was not significant in any of the baseline Task Types but that it reached significance in all IVIS Task Types except the Calling and Dialing task. Task Type Contrasts suggest that drivers found the nBack task to be the most difficult, followed by the SuRT task, then the Navigation Entry and Audio Entertainment tasks, then the Calling and Dialing and Text Messaging tasks, followed by the Single Task. Task Type Contrasts by Age Cohort suggest that the effect of Age Cohort was somewhat larger in the Audio Entertainment task than the SuRT task but that the Age Cohort effect was otherwise indistinguishable.

Inspection of the data reveals a relatively consistent effect of age across each of the in-vehicle tasks.

#### Age Cohort by Mode of Interaction

The interaction between Age Cohort and Mode of Interaction was also significant, χ^2^(1, 5) = 20.7, *p* > 0.001. Age contrasts found no effect of age in any of the tasks. Mode of Interaction contrasts found that the subjective evaluation of workload differed between the Modes of Interaction. Specifically, drivers felt that Auditory Vocal tasks were easier to complete than tasks completed using the Center Stack or Touchscreen displays. All of which were reported to be easier than the nBack task. Mode of Interaction contrasts by Age Cohort suggest that the magnitude of the Age Cohort effect was smaller in the SuRT task than several of the other task interactions.

#### Age Cohort by Mode of Interaction by Task Type

**FIGURE 5:**
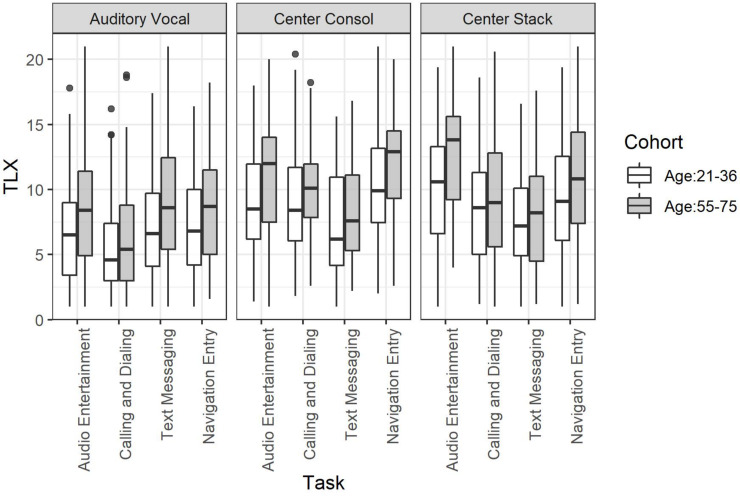
Task load index subjective workload for the full factorial of age cohort by mode of interaction by task.

The three-way interaction between each of these factors was not significant, χ^2^(1, 3, 2) = 7.52, *p* = 0.275. This lack of interaction is visible in Figure 5 where a main effect of age is apparent with highly consistent effects of Mode of Interaction and Task Type.

## Discussion

This research investigated the unique challenges that older drivers face when completing several common tasks using the In-Vehicle Information System (IVIS) of six 2018 vehicles. Prior research has shown that compared to younger drivers, older drivers exhibit greater difficulty dividing attention between tasks. This has been shown both in general laboratory tasks and in driving. How these generally reported differences are manifest in interactions with real-world vehicle technologies has not been well studied. This research provides additional insight into the unique challenges faced by older drivers as they interact with modern in-vehicle technologies, by addressing two sets of previously unanswered questions related to IVIS use.

Q1: Do IVIS interaction demands differ for older and younger drivers? If so, how?

Results suggest that, compared to younger drivers, older drivers experienced increased workload when interacting with IVIS. Older adults were slower to respond to the DRT stimuli (higher cognitive demand), were more likely to fail to respond to the forward LED (higher visual demand) and required more time to complete all tasks (increased exposure). Measures of cognitive, visual, and temporal demand for older and younger drivers indicated nearly identical patterns between all conditions (e.g., conditions that resulted in high workload for younger drivers also resulted in high workload for older drivers). However, measured workload for older drivers was consistently higher than for younger drivers.

Q2: Are some interfaces more difficult for older drivers to use? If so, why?

Older drivers had an especially difficult time maintaining visual attention to the forward roadway during secondary task interactions (as quantified by Hit Rate to the forward LED DRT stimulus – See Strayer et al., 2017), especially when completing IVIS tasks. IVIS task interactions are demanding in general but especially so for older drivers. Older drivers may benefit from interface designs that promote their continued visual attention on or near the forward roadway (e.g., careful placement of physical controls and dials, screen placement in-line with forward vision, use of voice controls, etc.).

### Summary of Results

#### Task Completion Time

Task Completion Time is an important facet of driver workload as it represents exposure. All other demands being equal, tasks that require longer to complete will result in greater distraction potential (see Strayer et al., 2015). When compared to younger drivers, older drivers required more time to complete tasks in all experimental conditions. Some noteworthy differentiation occurred between Task Type and Mode of Interaction.

• On average, both younger and older drivers completed the Audio Entertainment and Calling and Dialing Task Types in less than the 24-s time reference. Navigation Entry and Text Messaging Task Types each required significantly more than 24-s for both younger and older drivers.
• Tasks were completed more quickly using the center stack display by both age groups. Older drivers, on average, required more than 24-s to complete the Auditory Vocal and Center Console tasks.

#### Reaction Time to the DRT

Reaction Time to the DRT is a reliable and valid indicator of cognitive demand (cf. International Organization for Standardization, 2015). Interestingly, Reaction Time to each of the tasks was insensitive to differences in Task Type, and Mode of Interaction. Reaction Time did, however, greatly differ between the Single Task baseline and any of the other tasks and was longer for older drivers. The stability of Reaction Time during IVIS interactions regardless of Task Type and Mode of Interaction, suggests that at least with some tasks, cognitive demand remains constant throughout task engagement. Our analysis found that:

• Older drivers were slower than younger drivers, across all Task Types.
• Older drivers were slower than younger drivers for all Modes of Interaction.

#### Hit Rate to the DRT

Hit Rate to the DRT indicated that older drivers had a more difficult time dividing visual attention between driving and secondary tasks. Similar to Reaction Time, the effect of Age Cohort was consistent for each of the four Task Types, and across each of the three Modes of interaction. Our analysis found that:

• Older drivers hit rate was greatly reduced across all Task Types compared with younger drivers.
• Older drivers were less likely to respond to the forward LED across each of the three Modes of Interaction.

#### Subjective Measures

• All drivers found the Calling and Dialing and Text Messaging Task Types to be less demanding than the Navigation Entry and Audio Entertainment Task Types. Overall, older drivers reported IVIS task interactions to be more demanding than younger drivers.
• Both older and younger drivers reported that voice commands were easier to use than the Center Console or Center Stack controls. Older drivers reported that all Modes of Interaction were more demanding than reported by younger drivers.
• Comments from both groups were primarily negative in tone. The similarity in comments made by drivers emphasizes a need for improvement of these in-vehicle systems for all drivers across the age range.

### Further Discussion

The time required to complete tasks is a simple and effective way to evaluate general task demands. This has been noted by other researchers when applied to visual-manual interactions with IVIS systems (e.g., Green, 1999). We found that all tasks imposed cognitive and visual demands. Not surprisingly however, hit rates to the forward LED were even lower for tasks that diverted driver’s eyes from the road. Thus, an assessment of task completion time with a measure of visual attention may sufficient to understand driver workload when completing the discrete tasks with IVIS.

Findings from this research suggest that workload estimates derived from younger drivers may underestimate the workload experienced by older drivers. In the Visual Manual Distraction Guidelines published by National Highway Transportation Safety Administration [NHTSA] (2013), a participant sampling strategy that includes drivers from 4 age groups is recommended. Given the consistent performance differences between younger and older drivers, we recommend that future testing give higher priority to evaluating older users. Systems that older adults find easy to use will also be usable by younger adults; however, the converse may not always be the case.

A logical and potentially incorrect generalization of these findings would be to assume that poorly performing Task Types or Modes of Interaction would result in increased on-road distraction. While this may be true, it may also be the case that drivers naturally refrain from activities that are complex, error prone, or slow to complete. Frustration arising from these tasks may cause drivers to seek out simpler ways of IVIS interaction. For example, voice recognition systems in vehicles show promise, however, driver usage of these systems continues to be low (Viita, 2014). The reason being that they often require the use of precise keywords spoken in a very specific, and rigid order. The result may be an interaction that is more complex, frustrating, and distracting than the same action completed using the touchscreen on the center-stack.

Results from this evaluation should, therefore, be interpreted as a measure of the user experience, or distraction potential (Ranney et al., 2009; Lee and Strayer, 2004), and not necessarily a reflection of the level of on-road distraction that would be expected from these Task Types and Modes of Interaction. Paradoxically, it may be the case that the most difficult and demanding systems evaluated in this research are also the least likely to result in driver distraction because they are not used. Furthermore, there is the possibility that the in-vehicle information systems that are the most cumbersome to use may ultimately result in users abandoning the IVIS in lieu of their personal cell phone to achieve the same tasks. This captures what has been described as the Usability Paradox (Lee and Strayer, 2004) wherein distraction may increase with usability. Likewise, poorly designed systems may discourage use and therefore decrease distraction potential overall. Complex user requirements may pose unnecessary system-based barriers, which could result in circumstances where older drivers are faced with no good options.

### Design Recommendations

Compared to younger drivers, older drivers in this research exhibited slower Reaction Time, decreased Hit-Rate, longer Task Completion Time, and reported higher task demand when interacting with IVIS. These findings suggest that, at a minimum, older drivers should be included in a Universal Design validation as their interactions with vehicle technologies may significantly differ from that of younger drivers (Czaja et al., 2009). Relevant principles of Universal Design for vehicle manufacturers include Equity, Flexibility, Simplicity, Perceptibility, Error Recovery, and Accessibility (Farage et al., 2012). These principles may provide a framework for improvement of IVIS design. Clearly, an emphasis on simplicity would benefit drivers of all ages (Farage et al., 2012).

Both older and younger drivers exhibited difficulty dividing attention between tasks presented on the touch screen and the forward roadway. Data from this study suggests that a center console interactions are especially cumbersome for older drivers. Care should be taken to help drivers maintain their visual attention on the forward roadway without introducing unnatural interfaces that may cause interference with safe driving. While voice commands may help to reduce many of the potential problems of other interface types, they will only be used by drivers if the systems accurately process requests in a timely fashion. Even so, auditory vocal interactions imposed a relatively high level of cognitive demand on drivers. No interface is demand free and all interactions with vehicle technologies should be carefully considered and restricted when reasonable.

## Conclusion

This research investigated the challenges faced by younger and older drivers as they completed several common tasks using the In-Vehicle Information System (IVIS) of a representative sample of six 2018 vehicles. Compared to younger drivers, older drivers exhibited significant increases in cognitive and visual workload when completing IVIS tasks. Older drivers had difficulty dividing their visual attention between IVIS tasks and the forward roadway. In some cases, older drivers responded to fewer than 25% of LED illuminations presented on the forward windscreen.

Older drivers also required significantly more time than younger drivers to complete all task interactions. An analysis of subjective workload found that drivers were generally aware of task demands but may have underestimated their actual workload, as quantified in the other measures. Comments provided by drivers after each task interaction suggested that both older and younger drivers shared similar concerns about the experience of modern IVIS. Results from this research suggest that current versions of IVIS are demanding and difficult to use, especially for older drivers. For drivers to fully realize the potential benefits of current and future vehicle technologies, a renewed focus on accessible design is required.

## Terms and Nomenclature

**Table:** 

*Detection Response Task (DRT)*	The DRT is an International Standards Organization protocol (International Organization for Standardization, 2015) for measuring attentional effects of cognitive demand in driving. In this research, a vibrotactile device emitted a small vibration stimulus, similar to a vibrating cell phone or an LED light stimulus changing color from orange to red. These changes cued the participant to respond as quickly as possible by pressing the microswitch attached to a finger against the steering wheel. DRT reaction time increases and hit rate decreases as the workload of the driver increases.

*In-vehicle information system (IVIS)*	The collection of features and functions in vehicles that allow motorists to complete tasks unrelated to driving while operating the vehicle. In this report, the terms IVIS and system are used interchangeably. The IVIS features we tested involved up to four Task Types (see below) and up to three Modes of Interaction (see below).

*Modes of Interaction*	The way a user interacts with an IVIS to perform a task. Modes of Interaction were categorized into three types: Voice Commands, Center Stack, and Center Console. In this report, Mode and Mode of interaction is used interchangeably.

*NASA TLX*	A questionnaire-based metric assessing the subjective workload of the driver. The TLX assesses mental demand, physical demand, temporal demand, performance, effort, and frustration.

*nBack task*	The nBack task presented a prerecorded series of numbers ranging from 0 to 9 at a rate of one digit every 2.25 seconds. Participants were instructed to say out loud the number that was presented two trials earlier in the sequence. The nBack task places a high level of cognitive demand on the driver without imposing any visual/manual demands and was used as a high workload reference task.

*Primary driving task*	Activities that the driver must undertake while driving including navigating, path following, maneuvering, and avoiding obstacles.

*Reference task*	A task used for the purpose of comparing different tests or test results across vehicles or systems.

*Single-task baseline*	When the driver is performing the primary driving task (i.e., driving) without the addition of workload imposed by IVIS interactions.

*Secondary-task*	A non-driving related additional task.

*SuRT task*	The variant of the Surrogate Reference Task (SuRT, ISO TS 14198) used in this report required participants to use their finger to touch the location of target items (larger circles) presented in a field of distractors (smaller circles) on an iPad Mini tablet computer that was mounted in a similar position in all the vehicles. The SuRT task places a high level of visual/manual demand on the drivers because they must look at and touch the display to perform the task. The SuRT task served as a reference for the visual/manual demands associated with performing IVIS interactions.

*Task completion time*	The time to complete a task. Task completion time was defined as the time from the moment participants first initiated an action to the time when that action had terminated, and the participant said, “done.” When assessed using the visual occlusion methodology, the NHTSA guidelines provide an implicit upper limit of
	24 seconds of total task time. While originally intended for visual/manual tasks, these guidelines provide a reasonable upper limit for task durations of any Mode or Task Type.

*Task Type*	Tasks were categorized into one of four Task Types: Audio Entertainment, Calling and Dialing, Text Messaging, and Navigation, depending on vehicle capabilities. These Task Types were completed via different Modes equipped in each vehicle for each interaction.

*Visual demand*	The visual workload associated with the performance of a task. This would include the structural interference associated with taking the eyes off the forward roadway as well as the central interference in visual processing that arises from cognitive demand. In this report, we refer to the visual demand associated with performing IVIS tasks with different Modes of Interaction when the vehicle is in motion.

*Visual reference task*	A variant of the SuRT task (see above) served as the visual reference task in the current research.

*Voice Commands*	The Voice Commands method in which users communicate with the IVIS via voice recognition and structured commands. Voice Commands are aimed toward hands free interactions but may incorporate some visual manual interactions such as using steering wheel controls for activation. Voice Commands are one of the three Modes of Interaction evaluated in this research.

*Workload*	The aggregate of cognitive, visual, and manual demands on the driver. A motorist’s workload reflects a combination of demands from the primary task of driving and any secondary tasks performed by the driver. The terms demand and workload are used interchangeably in this report and we develop separate metrics for cognitive workload and visual workload.

	

## Data Availability Statement

The datasets for this study can be found on the Utah Center for Driver Safety and Technology github repository https://github.com/utahcdst/Aging-Report-Frontiers.

## Ethics Statement

The studies involving human participants were reviewed and approved by The University of Utah Institutional Review Board (IRB). The patients/participants provided their written informed consent to participate in this study.

## Author Contributions

All authors listed have made a substantial, direct and intellectual contribution to the work, and approved it for publication.

## Conflict of Interest

The Detection Response Task (DRT) devices used in this research were assembled by Red Scientific Inc., a company which is owned and operated by JC. Device specifications used for DRT assembly adhered to ISO 17488, as did all data handling and cleaning procedures.

The remaining authors declare that the research was conducted in the absence of any commercial or financial relationships that could be construed as a potential conflict of interest.

## Abbreviations

AAAFTS: AAA Foundation for Traffic Safety
DRT: Detection Response Task
FHWA: Federal Highway Administration
HMI: Human-Machine Interface
IRB: Institutional Review Board
ISO: International Organization for Standardization
IVIS: in-vehicle information system
LCD: Liquid-Crystal Display
LED: Light-Emitting Diode
MVA: Motor Vehicle Accidents
NHTSA: National Highway Traffic Safety Administration
OEM: Original Equipment Manufacturer
OS: Operating System
PFC: Prefrontal Cortex
SAE: Society of Automotive Engineers
SuRT: Surrogate Reference Task
TEORT: Total Eyes-Off-Road Time
USB: Universal Serial Bus.
