# Supplementary material for: Age-Related Differences in the Cognitive, Visual, and Temporal Demands of In-Vehicle Information Systems
Source: Front Psychol. 2020 Jun 3;11:1154. doi: 10.3389/fpsyg.2020.01154 (PMC7283540; doi:10.3389/fpsyg.2020.01154)
Supplement: Supplementary file 1 [file Data_Sheet_1.pdf]

Appendix A

A. Audio Entertainment Tasks - *Standardized Task List*

- Radio frequency tuning (AM, FM)
- iPod content (songs, artists, albums, genres)
- This task is complete when the audio entertainment has successfully been changed

1. Choose a **Jazz song** from the **iPad**
2. Play **1020 AM**
3. Tune the **radio** to **98.5 FM**
4. Listen to the **song "99 Red Balloons"**
5. You want to hear a song by the **band "Nirvana"**
6. Change the **radio** to your favorite **FM station**
7. Play a **song** in the **"Metal" genre**
8. Let's hear the **song "I'm Gonna Be (500 Miles)"**
9. You want to hear one of **your favorite AM stations**
10. Tune **AM 1540**
11. **89.1**
12. Tune to **1240**
13. **iPod** play **album "Storyline"**
14. **AM 1160**
15. Play a song by the **artist "Eminem"**
16. Play the **album "Homesick"**
17. **90.1**
18. You want to **play "Johnny Cash"** songs
19. **Radio 1630**
20. You want to play the **song "Riptide"** from the **iPod**
21. Switch to **the artist "Louis Armstrong"**
22. Play the **"Alternative" genre**
23. Change the **genre to "Reggae"**
24. **Radio** tune to **97.1 FM**
25. You want to hear the **artist "Hunter Hayes"**
26. Change the music to the **song "Three Little Birds"**
27. Listen to **FM 99.5**

Vehicle	Audi A6		Cadillac CT6		Lincoln Navigator		Mazda CX-5		Nissan Pathfinder		Volvo XC90	
Mode of Interaction	CS	VC	CC	VC	CS	VC	CC	VC	CS	VC	CS	VC
Audio Entertainment	A	A	A	A	A	A	A	A	A	A	A	A
Calling and Dialing	B	B	B	B	B	B	B	B	C	B	B	B
Navigation	D	D	D	D	E	D	F	F	G	D	D	D
Text Messaging	H	H	I	-	J	J, K	H	H	H	H	-	H*

B. Calling & Dialing Tasks - *Standardized Task List*

- Participant calls contacts (cell phone, work).
- Dials numbers (participant's own phone number, 801-555-1234).
- Task is complete once call has been successfully ended.

1. **Jack Olsen** would like you to call him on his **cell phone**
2. You need to call **8"OH"1-555-1234**
3. Place a call to the contact **Willow Brooks**
4. Try to reach **Brad Peterson**
5. Enter **8"ZERO"1-555-1234**
6. You can't find **your phone**. Call it to find it.
7. Ring **Felicity Gomez's office**
8. Enter your **own number**
9. You missed a call from **Oliver Reed**
10. Telephone **8"OH"1-555-1234**
11. **Violet Wheeler** is waiting to hear back from you on her **mobile**
12. Dial your **own number**
13. Give **Phil Potter** a call back **at work**
14. Give **8"ZERO"1-555-1234** a call
15. Try **Helen Harold** on her **business number**
16. Call your **own phone**
17. **8"OH"1-555-1234**
18. **Bethany Swan, cell phone**
19. Telephone **Jennifer Long**
20. You need to talk to **Yolanda Chavez**
21. Dial **Tanya Henry**
22. Call **Andrew Fink's mobile** back

Vehicle	Audi A6		Cadillac CT6		Lincoln Navigator		Mazda CX-5		Nissan Pathfinder		Volvo XC90	
Mode of Interaction	CS	VC	CC	VC	CS	VC	CC	VC	CS	VC	CS	VC
Audio Entertainment	A	A	A	A	A	A	A	A	A	A	A	A
Calling and Dialing	**B**	**B**	**B**	**B**	**B**	**B**	**B**	**B**	C	**B**	**B**	**B**
Navigation	D	D	D	D	E	D	F	F	G	D	D	D
Text Messaging	H	H	I	-	J	J, K	H	H	H	H	-	H*

C. Calling & Dialing Tasks - *Standardized Task List*

- Participant calls contacts (cell phone, work).
- Dials numbers (participant's own phone number, 801-555-1234).
- The task is complete once the call has been successfully ended.

1. **Jack Olsen** would like you to call him on his **cell phone**
2. Place a call to the contact: **Willow Brooks**
3. Try to reach **Brad Peterson**
4. Ring **Felicity Gomez's cell**
5. You missed a call from **Oliver Reed**
6. **Violet Wheeler** is waiting to hear back from you on her **mobile**
7. Give **Phil Potter** a call back
8. Try **Helen Harold** on her **mobile number**
9. **Bethany Swan, cell phone**
10. Telephone **Jennifer Long**
11. You need to talk to **Willow Brooks**
12. Dial **Brad Peterson**
13. Call **Jack Olsen mobile** back
14. You need to call **Jennifer Long**
15. Place a call to **Helen Harold**
16. You can't reach **Bethany Swan**. Call them again.
17. You need to reach **Oliver Reed**
18. Telephone **Phil Potter**
19. Dial **Felicity Gomez mobile**
20. Give **Violet Wheeler** a call

Vehicle	Audi A6		Cadillac CT6		Lincoln Navigator		Mazda CX-5		Nissan Pathfinder		Volvo XC90	
Mode of Interaction	CS	VC	CC	VC	CS	VC	CC	VC	CS	VC	CS	VC
Audio Entertainment	A	A	A	A	A	A	A	A	A	A	A	A
Calling and Dialing	B	B	B	B	B	B	B	B	C	B	B	B
Navigation	D	D	D	D	E	D	F	F	G	D	D	D
Text Messaging	H	H	I	-	J	J, K	H	H	H	H	-	H*

D. Navigation Tasks - *Adjustments for Vehicle Capabilities*

- Participant sets the destination to a point of interest that best fits the task goal.
- The task is complete once the participant successfully cancels route guidance.

1. **(Gas)** Fill up at the closest gas station.<br>2. **(Library)** Your library book is overdue. Let's return it at the closest library.<br>3. **(Italian Restaurant)** You're headed out for some Italian food at nearby restaurant.<br>4. **(Coffee shop)** Grab yourself a cup of coffee from the closest Starbucks.<br>5. **(Grocery store)** You need some items from Whole Foods.<br>6. **(ATM\bank)** You need to get cash from a Wells Fargo bank.<br>7. **(Mexican Restaurant)** Find a Mexican restaurant nearest you.	8. **(Hospital)** Go visit your friend at the LDS Hospital.<br>9. **(Chinese Restaurant)** You're craving food from Panda Express.<br>10. **(Movie theater)** You're on your way to see a movie at the nearby theater.<br>11. **(Hotel/Motel)** Drive to the nearest lodging to stay the night.<br>12. **(Post office)** You have a package to drop off at the closest Post Office.<br>13. **(Museum)** Go check out the new exhibit at the Utah Museum of Natural History.<br>14. **(Shopping Center)** Go pick out some new clothes at a nearby shopping mall.

Vehicle	Audi A6		Cadillac CT6		Lincoln Navigator		Mazda CX-5		Nissan Pathfinder		Volvo XC90	
Mode of Interaction	CS	VC	CC	VC	CS	VC	CC	VC	CS	VC	CS	VC
Audio Entertainment	A	A	A	A	A	A	A	A	A	A	A	A
Calling and Dialing	B	B	B	B	B	B	B	B	C	B	B	B
Navigation	**D**	**D**	**D**	**D**	E	**D**	F	F	G	**D**	**D**	**D**
Text Messaging	H	H	I	-	J	J, K	H	H	H	H	-	H*

E. Navigation Tasks - *Standardized Task List*

- Participant sets the destination to a point of interest that best fits the task goal.
- The task is complete once the participant successfully cancels route guidance.

1. **(Gas)** Fill up at the closest gas station.
2. **(Library)** Your library book is overdue. Let's return it at the closest library.
3. **(Restaurant)** You're headed out for some food at a nearby restaurant.
4. **(Coffee shop)** Grab yourself a cup of coffee from the closest coffee shop.
5. **(Grocery store)** You need some items from a nearby grocery store.
6. **(ATM\bank)** You need to get cash from a nearby bank or ATM.
7. **(Restaurant)** Find another restaurant nearby.
8. **(Hospital)** Go visit your friend at a nearby hospital.
9. **(Cafe)** You're craving food from a nearby cafe.
10. **(Movie theater)** You're on your way to see a movie at the nearby theater.
11. **(Hotel/Motel)** Drive to the nearest lodging to stay the night.
12. **(Post Office)** You have a package to drop off at the closest Post Office.
13. **(Museum)** Go check out the new exhibit at a nearby museum.
14. **(Shopping Center)** Go pick out some new clothes at a nearby shopping mall.

Vehicle	Audi A6		Cadillac CT6		Lincoln Navigator		Mazda CX-5		Nissan Pathfinder		Volvo XC90	
Mode of Interaction	CS	VC	CC	VC	CS	VC	CC	VC	CS	VC	CS	VC
Audio Entertainment	A	A	A	A	A	A	A	A	A	A	A	A
Calling and Dialing	B	B	B	B	B	B	B	B	C	B	B	B
Navigation	D	D	D	D	E	D	F	F	G	D	D	D
Text Messaging	H	H	I	-	J	J, K	H	H	H	H	-	H*

F. Navigation Tasks - *Adjustments for Vehicle Capabilities*

- Participant sets the destination to a point of interest that best fits the task goal.
- The task is complete once the participant successfully cancels route guidance.

1. **(Gas)** Fill up at the closest gas station.
2. **(Library)** Your library book is overdue. Let's return it at the closest library.
3. **(Italian Restaurant)** You're headed out for some Italian food at nearby restaurant.
4. **(Cafe)** Grab yourself a cup of coffee from the closest Starbucks.
5. **(Grocery store)** You need some items from Fresh Market.
6. **(ATM\Bank)** You need to get cash from a Wells Fargo bank.
7. **(Mexican Restaurant)** Find a Mexican restaurant nearest you.
8. **(Hospital)** Go visit your friend at the LDS Hospital.
9. **(Chinese Restaurant)** You're craving food from Panda Express.
10. **(Movie theater)** You're on your way to see a movie at the nearby theater.
11. **(Hotel/Motel)** Drive to the nearest lodging to stay the night.
12. **(Post office)** You have a package to drop off at the closest Post Office.
13. **(Museum)** Go check out the new exhibit at the Utah Museum of Natural History.
14. **(Shopping Center)** Go pick out some new clothes at a nearby shopping mall.

Vehicle	Audi A6		Cadillac CT6		Lincoln Navigator		Mazda CX-5		Nissan Pathfinder		Volvo XC90	
Mode of Interaction	CS	VC	CC	VC	CS	VC	CC	VC	CS	VC	CS	VC
Audio Entertainment	A	A	A	A	A	A	A	A	A	A	A	A
Calling and Dialing	B	B	B	B	B	B	B	B	C	B	B	B
Navigation	D	D	D	D	E	D	**F**	**F**	G	D	D	D
Text Messaging	H	H	I	-	J	J, K	H	H	H	H	-	H*

G. Navigation Tasks - *Standardized Task List*

- Participant sets the destination to a point of interest that best fits the task goal.
- The task is complete once the participant successfully cancels route guidance.

1. **(Gas)** Fill up at the closest gas station.
2. **(Rest Stop)** You left your wallet at the Jordanelle Rest Stop. Go retrieve it.
3. **(Restaurant)** You're headed out for some dinner at nearby restaurant.
4. **(Gas Station/Restaurant)** Grab a beverage from somewhere nearby to hydrate yourself.
5. **(Hotel/Motel)** Go check in at the closest motel for the night.
6. **(ATM\bank)** You need to get cash from a Wells Fargo bank.
7. **(Restaurant)** You are hungry for a sandwich, go to a place to satisfy your craving.
8. **(Hospital)** Go visit your friend at a Hospital.
9. **(Gas)** Go check your tire pressure at a nearby gas station.
10. **(Hotel/Motel)** Drive to the nearest lodging to stay the night.
11. **(Fire Station)** Attend the free CPR training at the nearby fire station.
12. **(ATM)** Deposit a check at a nearby ATM.
13. **(Rest Stop)** Make sure to see the sights at Coalville rest stop.

Vehicle	Audi A6		Cadillac CT6		Lincoln Navigator		Mazda CX-5		Nissan Pathfinder		Volvo XC90	
Mode of Interaction	CS	VC	CC	VC	CS	VC	CC	VC	CS	VC	CS	VC
Audio Entertainment	A	A	A	A	A	A	A	A	A	A	A	A
Calling and Dialing	B	B	B	B	B	B	B	B	C	B	B	B
Navigation	D	D	D	D	E	D	F	F	G	D	D	D
Text Messaging	H	H	I	-	J	J, K	H	H	H	H	-	H*

H. SMS - *Standardized Task List (Read and Send)*

- Participant reads or listens to text messages and responds with system-specific predetermined messages.*
- The task is complete once the participant successfully sends a message.

1. Read out the message from **Cam Whitman**. Please respond.
2. Read and reply to the text from **Scarlet Miles**
3. What did **Duncan Redford** send you? Send your answer.
4. **Maggie Carter** just messaged you. What should you send back?
5. What did **Connie Motts** say? Reply to her.
6. Find a message from **Andy Cameron**. Reply.
7. **Jane Evans** sent you a new text. Send something back.
8. Read the text from **Duncan Redford** and respond to it.
9. What did **Maggie Carter** send you? Send a text back.
10. Read and respond to the text from **Scarlet Miles**
11. New message from **Cam Whitman**. How do you reply?
12. What did **Andy Cameron** send you? Answer him.
13. You need to read and reply to **Jane Evan's** message.
14. Read out the message from **Maggie Carter.** Send your reply.
15. What did **Connie Motts** send you? Text her back.
16. How do you respond to the text from **Duncan Redford**?
17. What does the message from **Jane Evans** say? Respond.
18. Read and then reply to the new message from **Connie Motts.**

Vehicle	Audi A6		Cadillac CT6		Lincoln Navigator		Mazda CX-5		Nissan Pathfinder		Volvo XC90	
Mode of Interaction	CS	VC	CC	VC	CS	VC	CC	VC	CS	VC	CS	VC
Audio Entertainment	A	A	A	A	A	A	A	A	A	A	A	A
Calling and Dialing	B	B	B	B	B	B	B	B	C	B	B	B
Navigation	D	D	D	D	E	D	F	F	G	D	D	D
Text Messaging	**H**	**H**	I	-	J	J, K	**H**	**H**	**H**	**H**	-	**H***

**The 2018 Volvo XC90 allows for free dictation. Replies are not limited to predefined messages, meaning participant responds with genuine message content.*

I. SMS - *Adjustments for Vehicle Capabilities (Read Only)*

- Participant reads or listens to text messages and responds with system-specific predetermined messages.
- Task is complete once the participant successfully sends a message

1. Read out the message from **Cam Whitman**. Please respond.
2. Read and reply to the text from **Connie Motts**
3. What did **Duncan Redford** send you? Send your answer.
4. **Maggie Carter** just messaged you. What should you send back?
5. What did **Connie Motts** say? Reply to her.
6. Find a message from **Cam Whitman**. Reply.
7. **Maggie Carter** sent you a new text. Send something back.
8. Read the text from **Duncan Redford** and respond to it.
9. What did **Connie Motts** send you? Send a text back.
10. Read and respond to the text from **Maggie Carter**
11. New message from **Cam Whitman**. How do you reply?
12. What did **Duncan Redford** send you? Answer him.
13. You need to read and reply to **Connie Motts's** message.
14. Read out the message from **Maggie Carter.** Send your reply.
15. What did **Connie Motts** send you? Text her back.
16. How do you respond to the text from **Duncan Redford**?
17. What does the message from **Cam Whitman** say? Respond.
18. Read and then reply to the new message from **Connie Motts.**

Vehicle	Audi A6		Cadillac CT6		Lincoln Navigator		Mazda CX-5		Nissan Pathfinder		Volvo XC90	
Mode of Interaction	CS	VC	CC	VC	CS	VC	CC	VC	CS	VC	CS	VC
Audio Entertainment	A	A	A	A	A	A	A	A	A	A	A	A
Calling and Dialing	B	B	B	B	B	B	B	B	C	B	B	B
Navigation	D	D	D	D	E	D	F	F	G	D	D	D
Text Messaging	H	H	I	-	J	J, K	H	H	H	H	-	H*

J. SMS - *Adjustments for Vehicle Capabilities (Read Only)*

- Participant listens to text messages and responds with system-specific predetermined messages.
- The task is complete once the participant successfully reads the desired message aloud.

1. Read out the message from **Cam Whitman**.
2. Read the text from **Scarlet Miles**
3. What did **Duncan Redford** send you?
4. **Maggie Carter** just messaged you.
5. What did **Connie Motts** say?
6. Find a message from **Andy Cameron**.
7. **Jane Evans** sent you a new text.
8. Read the text from **Duncan Redford**.
9. What did **Maggie Carter** send you?
10. Read the text from **Scarlet Miles**
11. New message from **Cam Whitman**.
12. What did **Andy Cameron** send you?
13. You need to read **Jane Evan's** message.
14. Read out the message from **Maggie Carter.**
15. What did **Connie Motts** send you?
16. How do you respond to the text from **Duncan Redford**?
17. What does the message from **Jane Evans** say?
18. Read the new message from **Connie Motts.**

Vehicle	Audi A6		Cadillac CT6		Lincoln Navigator		Mazda CX-5		Nissan Pathfinder		Volvo XC90	
Mode of Interaction	CS	VC	CC	VC	CS	VC	CC	VC	CS	VC	CS	VC
Audio Entertainment	A	A	A	A	A	A	A	A	A	A	A	A
Calling and Dialing	B	B	B	B	B	B	B	B	C	B	B	B
Navigation	D	D	D	D	E	D	F	F	G	D	D	D
Text Messaging	H	H	I	-	**J**	**J**, K	H	H	H	H	-	H*

K. SMS - *Adjustments for Vehicle Capabilities (Send Only)*

- Participant replies to the most recent text message with system-specific predetermined messages.
- The task is complete once the participant successfully sends the desired message.

1. Let **Duncan Redford** know you're going to be late.
2. **Duncan Redford** is asking if you want to go to the movies tonight.
3. **Duncan Redford** wants to go dancing tonight.
4. **Duncan Redford** texted you a funny joke.
5. Ask **Duncan Redford** to ask call you
6. **Duncan Redford asked where you are**.
7. Tell **Duncan Redford** you can call him soon
8. Tell **Duncan Redford** you are on your way.
9. **Duncan Redford** texted you a silly dad joke.
10. **Duncan Redford** has big news and is wondering if you can talk right now.
11. **Duncan Redford** dropped off your favorite cookies at your house.
12. **Duncan Redford** is wondering where you are.
13. **Duncan Redford** can pick you up from the airport next week.
14. Tell **Duncan Redford** to call you after **work**
15. Tell **Duncan Redford** you want to talk later
16. **Duncan Redford** wants to know if they can copy your homework.
17. **Duncan Redford** wants to know why you're not at the restaurant yet.
18. **Duncan Redford** says they will clean your car for you tonight.

Vehicle	Audi A6		Cadillac CT6		Lincoln Navigator		Mazda CX-5		Nissan Pathfinder		Volvo XC90	
Mode of Interaction	CS	VC	CC	VC	CS	VC	CC	VC	CS	VC	CS	VC
Audio Entertainment	A	A	A	A	A	A	A	A	A	A	A	A
Calling and Dialing	B	B	B	B	B	B	B	B	C	B	B	B
Navigation	D	D	D	D	E	D	F	F	G	D	D	D
Text Messaging	H	H	I	-	J	J, **K**	H	H	H	H	-	H*
